# New Medical Gazette

**Published:** 1859-08

**Authors:** 


### SURGICAL ITEMS.

### NEW MEDICAL GAZETTE.

Prof. Gustav C. E. Weber has issued at Cleveland, a monthly medical journal, of 32 pages, at one dollar per year. While several medical periodicals, among which, the *Montreal Chronicle*, the *Maine Medical Journal*, the *Louisville Medical Gazette*, have been induced to suspend for want of support, we cannot but honor the courage of one who assumes the responsibility of a new one.

Prof. Weber holds the chair of Surgery in the Cleveland Medical College, formerly so ably filled by the late lamented Prof. Ackley, and his journal gives promise of usefulness.

---

We believe that Prof. Hamilton has recently completed his work on fractures, and that it will soon be issued from the press.

